# Opposed Left and Right Brain Hemisphere Contributions to Sexual Drive: A Multiple Lesion Case Analysis

**DOI:** 10.1155/2003/123757

**Published:** 2003-01-01

**Authors:** Claude M. J. Braun, Mathieu Dumont, Julie Duval, Isabelle Hamel, Lucie Godbout

**Affiliations:** ^1^Centre de Neurosciences de la Cognition and Department of PsychologyUniversité du Québec à MontréalCanada; ^2^Department of PsychologyUniversité du Québec à Trois RivièresCanada

## Abstract

Brain topographical studies of normal men have have shown that sexual excitation is asymmetric in the brain hemispheres. Group studies of patients with unilateral epileptic foci and other studies of patients with unilateral brain lesions have come to the same conclusion. The present study reviewed previously published single case reports of patients with frank hypo or hypersexuality subsequent to a unilateral brain lesion. Hyposexual patients tended to have left hemisphere lesions (primarily of the temporal lobe), and hypersexual patients tended to have right hemisphere lesions (primarily of the temporal lobe) (*p* < 0.05). We interpret this double dissociation as part of a more general phenomenon of psychic tone similarly dissociated with regard to hemispheric control, including mood, psychomotor baseline, speech rate, and even immunity. The behavioral significance of this psychic tone is to modulate approach versus avoidance behavior.

## 1. Introduction

Human sexuality is, of course, modulated by the cortex of the cerebral hemispheres. Indeed, we engage in sexual fantasy and behavior under some degree of conscious representation and some degree of control. However, there is another aspect of human sexuality in which the brain hemispheres (though not necessarily just cortex) seem to be asymetrically involved, namely sexual tone, i.e., libido. One relatively easy way to study human sexuality from a neuroscience point of view is to break down sexuality into this simple bipolar dimension, hypo versus hypersexuality. Hyposexuality, for our purposes here, consists of low libido with an accordingly low level of sexual activity and hypersexuality consists of the opposite.

The temporal lobes are without contest those most intimately involved with regulation of sexual tone. Temporal lobe epilepsy strongly tends to produce hyposexuality [20,49]. Bilateral temporal lobe lesions markedly tend to produce hypersexuality [26,39,41,46,58], a symptom recognized as part of a larger syndrome named the Kluver-Bucy syndrome. Bilateral frontal lobe lesions also affect human sexual function [23,36,64] but less so than lesions of the temporal lobes. However, this portrait is not incompatible with the eventuality of a perhaps more subtle asymmetry of hemispheric contributions to sexual tone.

If one can demonstrate that left hemisphere lesions or dysfunctions differ in their effect from right hemisphere lesions or dysfunctions, then one can assume that there is some sort of special asymmetric contribution of the normal hemispheres, perhaps analogous to, or even causally linked to, other hemispheric asymmetries which are also manifest in the human species. In fact, there already exist several lines of evidence of asymmetries of hemispheric contributions to sexuality. Sexual excitement in men, for example, seems to be as-

ISSN 0953-4180/03/$8.00 © 2003 – IOS Press. All rights reserved sociated with asymmetric activation of the hemispheres as has been determined by metabolic imaging [55,57] and by EEG topography [16,45,60]. Epileptic paroxysmal orgasm seems to occur more frequently from right sided foci than left [15]. Interictal hyposexuality results more frequently from right foci than left ([19], but see [20], for inconclusive results in a very large sample of women epileptics) while hypersexuality seems to be slightly more often associated with left foci [9].

Several relevant studies of brain damaged patients from a single clinic have been published. Sandel and colleagues [47] found that right hemisphere lesions produced hypersexuality and left lesions hyposexuality. However, since the patients were all cases of head trauma, the localizing value of lesion analysis is doubtful. Goddess and colleagues [25], Kalliomaki and colleagues [28] and Kimura and colleagues [31] investigated stroke patients for libido. They all found significantly higher libido in the right hemisphere stroke group and lowered libido in the left hemisphere stroke group. Other studies have obtained inconclusive results however [11,50]. The only two investigations which found counter-results [1,17] excluded depressed patients. Since a third of stroke patients are depressed, and a large majority of these have left hemisphere lesions and depression and delibidinization are highly related in stroke patients [31], the two studies with counter-results can in fact be interpreted as supportive of those of Sandel, Godesss, Kalliomaki, Kimura and their respective colleagues. Of course, only a *relative* tendency for hypersexuality in right hemisphere stroke can be expected, considering that many of these patients may be very handicaped, hemiplegic, on antihypertensive medication, and may be very worried about having further strokes due to sex. Fisher [22] also observed no relation between lesion site and libido in brain tumor patients.

Studies of groups of patients with hemispheric lesions all share one characteristic: the patients manifest relatively subtle to not so subtle variations of sexual feelings and behaviors and are graded on a scale by the investigators, as would be normal subjects. A limitation of this approach is that because libido is being considered here as a continuously distributed variable, extreme manifestations, which could be labelled *hypo* or *hyper* sexuality, cannot be identified, and might in fact be very rare. The patient base required to prospectively assemble groups of hypo or hyper sexual patients might be prohibitive. There is however another approach to the problem: one can select patients on the basis of frank deviation of libido as well as a unilateral lesion from single case reports published in the literature. Luckily, a multiple case review of published cases can now provide a sufficient number of such cases for hypothesis testing. With the exponential growth of scientific publication, case reports of post-lesion psychiatric manifestations are becoming quite numerous. So much so that reviews of them can be designed to test hypotheses. We are not aware of such an investigation having been carried out to test the hypothesis of asymmetric hemispheric control of sexual drive.

The relevant evidence reviewed in the previous two paragraphs leads unambiguously to a simple model of double dissociation of hemispheric contribution to libido: the intact right hemisphere inhibits libido and the left enhances it. It was thus predicted, for the purpose of the present investigation (using meta-analysis of published case reports), that patients with unilateral focal brain lesions would manifest the following double dissociation: hypersexual patients would have right hemisphere lesions and hyposexual patients would have left hemisphere lesions. A second prediction was that hypo or hypersexuality ought to be caused by lesions of the temporal lobes more frequently than of any other lobe of the brain.

## 2. Method

This project is a multiple single case literature review analysis. We searched the literature for all single case reports of patients with unilateral focal hemispheric lesions leading to frank hypo or hypersexuality. Post-lesion depression was *not* an exclusion criterion, but any mention of mood disorder in any case is included in our data tables (tables 1 and 2). We present no cases with any mention of pre-lesion sexual problems. In order to understand the development of the “brain modules” in question, we set out to review the smallest possible unilateral lesions causing, earliest in development, syndromes comparable to those of adults. These cases would, ideally, present as few complications as possible (epilepsy, edema, hydrocephaly, etc.) to ensure that the symptoms are the effects of the lesion and not an artefact.

### *2.1. Limitations of the multiple case review approach*

There are numerous problems with the use of lesioned humans to study development and implementation of functions in the brain, of which we will review six. 1) Single case reports are typically published because they present some unusual trait. This can create sampling biases relative to the trait targeted for a multiple case review. In the same vein, determination of the trait under consideration (in this case, *hypo* or *hyper* sexuality) will necessarily vary from one case to the other and can at worst consist of mere anecdote. 2) Multiple case review analysis is only as good as the ingenuity and perseverance of the reviewer in locating relevant cases. A good survey of highly targeted neurogenic cases in the literature takes years of effort. 3) Not all functions are clearly organized in a few, well-circumscribed modules, as is language. Functional brain imaging studies, as a whole, suggest that mental operations are practically always multifocal [33]. A pathological condition may thus be associated with several lesion sites. Individual variations in brain morphology, but also in organisation of implemented cerebral functions, cause a wide array of alternative lesion sites responsible for a particular condition. 4) The injured brain can attempt relocation of functions in the other, undamaged hemisphere, especially if the lesion occurs early enough in development. Relocalisation can even be attempted in the injured hemisphere, near the lesioned area where the function would normally implement itself, especially in the case of small lesions [63]. It is conceivable that recovery is not as effective in these cases as in hemispherectomy. Relocalisation and recovery of functions in the child's brain likely cause an impairment profile greatly dissimilar to what is seen in the adult after a similar brain lesion. Due to relocalisation, other functions, not located in the damaged area, can be drawn aside (e.g., "crowded") to let the relocated function implement itself [56]. It could even be hypothesized that a focal cortical lesion could produce almost any psychiatric disorder in the child, due to this migration of functions. 5) Etiology is also a factor to be considered in the expression of psychiatric syndromes. Lesions caused by hemorrhage have far reaching acute effects such as edema, electrolyte disturbance, immune proliferation, etc. Certain mitotic lesions also have proximal electrical and metabolic irritative effects which can override the effect of tissue loss in the tumor area. This is well illustrated by Filley and Kleinschmidt (1995) who reported alleviation of psychiatric symptoms in all five of their operable neoplasic cases by surgical removal of the tumor. Epilepsy accompanying a lesion may, or may not, override and even contradict lesion effects by irritating functional tissue, as a main cause for psychiatric symptoms. 6) One can never be certain that the disorders seen in brain-lesioned persons are causally linked to the lesion site, or even with the lesion at all, on a case by case basis. A psychiatric syndrome could have developed in the patient, whether or not a lesion was present. The lesion could also be a minor element that was missing for the pathology to reach threshold. The location of the lesion could be, in such a case, only moderately relevant to the pathological manifestation. It is thus important to determine, when possible, whether there is presence of psychopathology prior to the lesion or in the family pedigree. The relation between the locus of the lesion and the manifested syndrome must therefore be interpreted with careful attention to age, pre-lesion status, co-morbidity (ex: mood disorder), etiology, complications (particularly epilepsy), and the present study covered these issues systematically.

In short, a multiple case review analysis in behavioral neurology must be viewed for what it is worth, a heuristic.

### *2.2. Advantages of the multiple case review method*

The case reports drawn from the literature are in the public domain and can be cross checked. The factual and technical aspects of the review can thus be challenged, as can be the theoretical conclusions of the interpreter. Over time, the data base can be reused and upgraded as well. When a multiple case analysis yields a clear result, it gives ground to the behavioral neurology community to expend the considerable effort required for appropriate prospective investigations. These can then provide more definitive tests of theories suggested in heuristic form. In the present case, a prospective study would very carefully identify unilateral lesions, would quantify pre-lesion sexual status (subjective as well as objective) as well as post-lesion status, and would intricately measure relevant co-variables and potential confounds (i.e., psychic tone [see our discussion], aphasia, motor impairments, anosagnosia, etc.).

## 3. Results

Certain cells of the design used for testing the first hypothesis contained less than five replicates. Consequently, we selected the Fisher Exact Test (FET) to test the inference of non random distributions of lesion cases as a function of lesion side and type of sexual disorder. We note 6 left hemisphere and 1 right hemisphere lesion cases with hyposexuality. Conversely, we note 3 left hemisphere and 8 right hemisphere lesion cases with hypersexuality (FET, $p < 0.05$, 2 tailed).

Table 1
Published cases with hyposexuality resulting from a unilateral hemispheric lesion

| Age at onset and sex of patient | Localization of the lesion | Symptomatological considerations | Etiological considerations and complications | Reference (first author and date) |
|---|---|---|---|---|
| 40 Male | Left temporal | Hyposexuality, aggressive violent destructive rage attacks, depression | Glioblastoma, left sided EEG slowing | Uribe, 1986 |
| 46 Male | Left temporal | Hyposexuality, hair fetishism | Tumor, epilepsy | Ball, 1968 |
| 47 Male | Left temporal | Hyposexuality, aggressiveness, depression, memory defect | Epilepsy, temporal lobectomy with occasional seizures after surgery | Stevens, 1990 |
| 37 Male | Left occipito-temporal | Hyposexuality, visual hallucinations, agitation and aggressiveness | Infarct, slowing of EEG on left | Medina et al., 1977 |
| 20 Male | Left internal capsule | Hyposexuality, diaper fetishism, | Infarct, normal EEG | Bethell, 1974 |
| 50 Male | Left pons, midbrain, hypothalamus, thalamus | Hyposexuality, pedophilia, exhibitionism | Astrocytoma, no mention of EEG or seizures | Miller et al., 1986 |
| 44 Male | Right thalamus | Hyposexuality, apathy, lethargy, attention deficits, executive deficits | Lacunar infarct, diffuse slowing in the EEG | Van Der Werf et al., 1999 |

The temporal lobe was clearly more frequently involved (10 cases) than was any other lobe. As expected, the frontal lobe was the next most frequently involved (3 cases).

## 4. Discussion

Libido seems to be organized in the brain in a doubly dissociated manner: the normal right hemisphere probably inhibits libido and the normal left hemisphere probably enhances it, as indicated by numerous techniques including the lesion approach. We propose to explain this phenomenon by enlarging the focus from sexuality to the ensemble of non cognitive phenomena that are also doubly dissociated in the human brain hemispheres.

Hypersexuality is associated with post-lesion mania ([6,18,51], but see [48]), pseudomania and hyperlalia [36,37]. Mania, pseudomania [12] and hyperlalia [13] all occur significantly more often as a result of right hemisphere than left hemisphere lesions. Hyposexuality is associated with post-lesion depression ([31,53,61], but see [40]) and pseudodepression [62]. Post-lesion mutism is usually akinetic, such that hyposexuality seems futile to note. Buge and colleagues [14] described three cases of post-lesion mutism who appeared profoundly anhedonic. Though no mention was made of sexual behavior or libido, one readily supposes that there was no libido at all. Depression, pseudodepression [12] and akinetic mutism [13] occur more frequently after left hemisphere than right hemisphere lesions. The same associations between these sets of clinical syndromes occur in cases which are not post-lesional [27,43].

These associations suggest that there exist integrated phenomena which together consist of baseline *psychic tone*. This "tone" includes motor, moral, emotional, language and sexual dimensions, and these are reflected in, of course, (and could perhaps be partially "caused" by) intellectual representations. Psychic tone may be "cold", "down", or "blue" or "exhausted" or "dull" or "flat" or "constricted". On the other hand it may be "hot", "high" , or "pink, or "excited" or "sharp" or "soaring" or "expansive". The two hemispheres of the brain exert opposed modulation on this ensemble of these "tones", in a coherent manner. A concept which encompasses all of these "tones" without surpassing their boundaries, i.e., which delineates a necessary and sufficient definition, is the *approach-avoidance* disposition. Even the immune system is clearly lateralized in the human hemispheres, with left hemisphere lesions producing immunosuppression and right lesions immunofacilitation [29,30,54]. Depression is associated with an increased risk for immunosuppression [42] while mania is associated with immunofacilitation [44]. We propose that the two hemispheres play opposed roles in the alarm or "stress" response, particularly in its *approach-avoidance* aspect. There are situations where it is important for the sexual response to be inhibited, situations of great danger (real or perceived or "felt"), i.e., that call for avoidance and flight or inaction. Inhibition or enhancement of the sexual response tone (the disposition to engage in sex), viewed in this simple neurodynamic and ecological context (which is, need we recall, only a small part of the overall determination of human sexuality), is part of the human stress

Table 2
Published cases with hypersexuality resulting from a unilateral hemispheric lesion

| Age at onset and sex of patient | Localization of the lesion | Symptomatological considerations | Etiological considerations and complications | Reference (first author and date) |
|---|---|---|---|---|
| 54 Female | Right frontal | Hypersexuality, mania | Meningioma, no mention of EEG or seizures | Starkstein et al., 1988 |
| 47 Female | Right temporal | Hypersexuality, hyperphagia, hyperlalia | Infarct, seizure activity | Monga et al., 1986 |
| 23 Male | Right temporal | Hypersexuality, mania | Arteriovenous malformation + emboly, no mention of EEG or seizures | Starkstein et al., 89 |
| 53 Male | Right temporo-frontal | Hypersexuality | Infarct, seizure activity | Monga et al., 1986 |
| 61 Male | Right occipital | Sexual disinhibition, apathy, indifference, withdrawal, depression | Infarct, no EEG or seizures reported | Price et al., 1983 |
| 31 Female | Right thalamic, hypothalamic | Hypersexuality, hyperphagia, logorrhea | Stroke, no mention of EEG or seizures | Miller et al., 1986 |
| 36 Female | Right thalamo-capsular | Hypersexuality, mania | Hematoma, normal EEG | Starkstein et al., 1988 |
| 10 Male | Right cingulate cortex | Public masturbation, sexual coprolalia, aggressiveness, agitation, compulsiveness | Astrocytoma and paroxysms (symptoms abated after ablation) | Angelini et al., 1980 |
| 69 Female | Left temporal | Hypersexuality (Kluver-Bucy syndrome), exibitionnism, propositioning, agitation | Oligodendroglioma, EEG slowing without epileptiform elements | Ghika-Schmid et al., 1995 |
| 55 Female | Left temporal | Hypersexuality, hyperphagia | Hemorrhage, no mention of EEG or seizures | Monga et al., 1986 |
| 33 Male | Left temporo-frontal | Hypersexuality, indecency (Kluver-Bucy syndrome), aphasia | Encephalitis, left temporal EEG slowing | Laurent et al., 1990 |

response. Hemispheric modulation of the human stress response consists of a dynamic balancing of opposed behavioral toning effects of the two hemispheres. Lesions of one or the other hemisphere thus pervert the human sex drive, as well as a host of other behavioral functions, in opposed manners. Finally, we predict that since the immune function [4,5] and psychomotor baseline [8,34] are asymmetrically organized in subhuman mammalian brain hemispheres, it will be found that effects on sexual behavior of unilateral lesions will produce similar dissociations in these species as well.
